# Correction: Direct determination of multiphoton absorption cross-sections by transient absorption spectroscopy

**DOI:** 10.1039/d6sc90153k

**Published:** 2026-08-10

**Authors:** Huajun He, Jia Wei Melvin Lim, Minjun Feng, Zengshan Xing, Tze Chien Sum

**Affiliations:** a Division of Physics and Applied Physics, School of Physical and Mathematical Sciences, Nanyang Technological University 21 Nanyang Link Singapore 637371 Singapore Tzechien@ntu.edu.sg

## Abstract

Correction for ‘Direct determination of multiphoton absorption cross-sections by transient absorption spectroscopy’ by Huajun He *et al.*, *Chem. Sci.*, 2025, **16**, 14924–14930, https://doi.org/10.1039/D5SC03392F.

The authors regret that there was a notational error in eqn (6) and the subsequent definition of *J*^(*n*)^. Eqn (6) is hereby corrected to the following:〈*N*(*t*)〉 = *σ*_n_·*g*^(*n*)^_p_/*τ*^*n*−1^·〈*J*(*t*)〉^*n*^·1/*n* (6)where *J*^(*n*)^ is defined as:*J*^(*n*)^ = *g*^(*n*)^_p_/*τ*^*n*−1^·〈*J*(*t*)〉^*n*^·1/*n*

This is solely a notational error and does not affect any of the reported experimental results, multiphoton absorption cross-section values, figures, data analysis, or the scientific conclusions of the paper. All numerical analyses were carried out using the correct expression.

The Royal Society of Chemistry apologises for these errors and any consequent inconvenience to authors and readers.

